# Development and validation of a robot social presence measurement dimension scale

**DOI:** 10.1038/s41598-023-28817-4

**Published:** 2023-02-19

**Authors:** Na Chen, Xiaoyu Liu, Yanan Zhai, Xueyan Hu

**Affiliations:** School of Economics and Management, Beijing University of Chemical Technology, Beijing, 100055 China

**Keywords:** Social behaviour, Human behaviour

## Abstract

Robots that can exhibit human-like behaviour, build social relationships with humans, and carry out social interaction that can be considered to have a social presence. Measuring the social presence of robots can provide an important basis for optimizing the design of robots’ human-like behaviour, but until now, there has been no research and discussion on the presence of robots from the perspective of social interaction. Based on related studies, such as the theory of the presence of interpersonal interaction society, the mechanism of network social presence measurement and human–computer interaction, this study constructs a theoretical model of robot social presence, and develops corresponding measurement scales with five dimensions, namely, the presence, attention distribution, interactive expression and information understanding, perceived emotional interdependence, and interaction behaviour perception. The 5-dimensional robot social presence model was finalized, and a 17-question questionnaire scale was developed. The validation of the model and the development of the scale includes one expert assessment (involving three experts), one user interview (involving five interviewees), and two questionnaire surveys (involving 172 valid questionnaires and 494 valid questionnaires respectively). The final model shows good reliability of the measurement questionnaire, high inter-factor correlation, high model fit, high internal consistency of the dimensions, high reliability of the scale, and high convergent validity of all dimensions. This study provides a theoretical basis for the study of robot social presence and human–robot collaboration and provides a reference measurement tool for social robot-related development and research.

## Introduction

The development of artificial intelligence technology has led to achievements in voice interaction, image recognition, motion interaction, and emotional expression. Robots express their feelings to users more smoothly^1^ and are involved in deeper interactions with humans, thus drawing attention to their social capabilities. Social robots are automatic procedural agents that can automatically generate content and actively participate in human social interactions^2^, have anthropomorphic social attributes and social behaviours, and can interact with users emotionally^3^, assist them in decision making^4^, provide companionship to reduce the number of users^5^, or provide companionship to reduce user isolation^5^. Social robots can be assistive tools for the elderly^6^ and people with physical or cognitive disorders^7,8^, help children with autism to improve their social skills^9^, and replace human customer service for customers^10^. For example, Estee Lauder has developed a chatbot, named No. 6 Mortimer, that can help customers select the ideal product (Estee^11^. The impact of robots on everyday life has increased, and there is literature on the level of intelligence and social functions of robots, but there is still a lack of analysis and research on their social attributes from a sociological perspective^12^.

Humans embody social attributes through interaction, i.e. the sum of human and other social relationships^13^. Robots can embody social attributes during human–computer interactions and can be regarded as members with social attributes by simulating human behaviour in their interactions. The functions social robots show, such as verbal communication, emotional expression, and helping behaviours^14,15^, can replace humans in satisfying users’ social-emotional needs, social needs, and care needs. The previous studies on the social attributes of robots are mostly based on the study of human social attributes, and social presence is considered one of the most important features of the social attributes of robots, which can be reflected by interactions with humans. Social presence theory^16^ suggests that a person’s sense of social presence is “the feeling of being with others in a coordinated environment”. Fong and colleagues^15^ suggests that if a robot is structurally and functionally similar to a human, then the robot can interact with humans as humans do. Kozan^17^ argues that social presence is closely related to the emotional connection between the interacting parties and that the emotional connection in the interaction affects the effectiveness of the interaction^18^; thus, the human perception of the robot’s social presence may influence the effectiveness of the human–robot interaction.

If people can perceive a higher level of social presence in robots, they can interact with the robot more naturally and smoothly, and the robot can provide better services. Therefore, academic investigation and measurement of the robots’ social presence attributes is important for both the expansion of human–robot interaction theory and for designing the human behaviour functioning of social robots. Several scholars have conducted studies on the social presence of robots. However, these studies have mainly explored the social presence of virtual characters in online environments, such as intelligent customer service and virtual social network users. There is still a lack of research on robots’ social presence, and a more comprehensive modelling framework and measurement tools have not been developed. Therefore, this study defines robots’ social presence, constructs theoretical models, identifies model dimensions, and develops the corresponding measurement scales based on the social presence of virtual characters and human social presence in online environments. This study extends the theory related to the social attributes of robots, provides measurement tools, and provides a theoretical basis and corresponding tools for the further development and improvement of the human-like behaviour functions of social robots.

## Literature review

### Research on social presence in interpersonal interaction

People have natural social attributes and have need of established, strengthened and maintained social relationships, and face-to-face behaviour and direct conversation can enhance such relationships^19^. Social relationships are central to interactions in modern social life, and direct social interaction is considered the most important factor in establishing and maintaining social interactions^20^. In interactions, people’s attention allocation was changed and focus of attention increased^21,22^, mean that they feel the social presence of the interaction object. Social presence^23^ means the degree to which one is perceived as a “real person” when communicating and the level of feeling connected to others^16^. Social presence, i.e. being together, can be defined as “others” or a sense of “being present with others in a coordination environment”. According to Zheng and colleagues^24^, social presence is “the degree to which private, warm, intimate, and social interactions are established with others in the environment”. Social presence is characterized by immediacy and intimacy. Immediacy refers to the ability of the interacting parties to communicate directly and respond to what is happening, while intimacy refers to the deepening of the relationship between individuals as cognition deepens and the interaction moves from the surface to the deeper level. Table 1 below is the relevant definition of social presence.

**Table 1:** A study of previous perspectives on social presence.

Main points	References
Social presence is “the degree of salience of others in mediated interactions and the resulting salience of interpersonal interactions”. The “gold standard” of social presence is face-to-face interaction, and the level of social presence is highest when two people are within reach of each other, especially when they are interacting on a task	^16^
Defines social presence as “other”	^25^, page 262
Social presence is the feeling of “being with others”. “Presence” and “live judgement” are the factors that influence the sense of social presence	^26^
Social presence is the individual’s moment-by-moment awareness of the simultaneous presence of another perceptual body, accompanied by a sense of participation with the other perceptual body	^27,28^, page 2
Social presence is the degree of salience of others in an interaction and the resulting salience of interpersonal relationships, including the degree of interaction perception, the degree of attention allocation, the ability to understand content and emotion, and the ability to form emotional and behavioural interdependence	^29^
The presence metric of a virtual environment is called “presence”	^30^
Social presence is the degree to which an individual establishes private, warm, and intimate social interactions with others in the environment	^24^
Social presence is the perception of the presence of others arising in a common context	^31^
Social presence refers to the quantity and quality of interpersonal communication in the environment as perceived by the individual	^32^
Social presence consists of 3 domains, first common presence, common factors and mutual awareness, followed by psychological involvement, including mutual attention, empathy, mutual understanding, and finally, behavioural involvement, including behavioural interaction, mutual aid, and dependent behaviour	^27,28^, page 4–5
There are three levels of social presence. The first tier is co-presence, which is a necessary but not a sufficient condition for a sense of social presence. The second level is the subjective perception level, which is an attempt to measure the psychobehavioural accessibility of another interactor. The third level is the intersubjective symmetry, which is an assessment of the symmetry between the internal and interacting objects	^26^
Social presence is caused by, but independent of, the presence of others	^33^, page 3
Social presence is “instant” and “intimate” in nature	^34^, page 213
Social presence is significantly associated with “immediacy” and “intimacy”, and direct interaction enhances the sense of social presence	^19^, page 151

Social presence has two properties. First, social presence represents connection. Heeter^25^ argues that social presence is a phenomenon in which two individuals are interrelated. Donath^35^ argues that there is a direct link between the level of social presence of others perceived by the user in the environment and the behaviour of the individual, which can be expressed through their behaviour, mental states and participation. The social presence of an individual is based on his or her having certain social relationships^36^. In social relations, people are able to perceive and judge the interaction objects that have a sense of presence^33^. Second, the social presence perceived by the individual is caused by the presence of others but it is the independently feeling by the individual^37^. Individuals rate the extent to which others have a sense of social presence based on their own feelings^38^. An individual is considered to have a social presence when he or she is aware of his or her simultaneous presence with other individuals at a given moment and feels the participation of that individual in the interaction.

Early scholars viewed social presence as one-dimensional, i.e. the “other” or “a feeling of being in a coordinated environment with others” felt by the individual (^25,16^, Biocca et al.^26^ further argue that social presence is the feeling of being with others. Social presence is influenced by immediacy^27,28^ and the significance of the interaction^16^. Individuals feel the social presence of others most strongly in the moment of interaction, and the deeper the interaction, the stronger this feeling.

Most current scholars consider social presence to be multidimensional, including Gunawardena^27,19^ and Nowak^39^, who argue that social presence contains three dimensions, i.e. superficial physical presence, the presence of mental states, and presence with behavioural involvement and interaction^25^. The first of these dimensions, superficial physical presence, i.e. the discovery and awareness of presence that coexists physically (physiologically) with the bodies of others, is a physical and objective presence caused by the inevitable meaning of human physiological attributes. When an individual senses the presence of others, the sense of co-presence in the same environmental space allows the individual to perceive the social presence of the other^27,28^. Zajonc^40^ argues that when in the presence of others, the individual’s attention changes in response to the presence of others, and the level of ego drive or arousal increases,thus, cognitive processing abilities change and lead to increased or decreased individual task performance.

The second dimension of mental state presence is the person’s subjective perception, which includes the expression and understanding of emotions, changes in attention allocation, and changes in mental states. Biocca^27,28^ proposed that when an individual senses the interaction intention or emotional state of an interacting object, the other person is considered to have a significant social presence because, according to the definition of social presence, if an individual realizes a significant degree of interaction or an interpersonal relationship is generated by an interaction, then it means that the individual has felt the corresponding degree of social presence^33^. Many previous social presence-related models include a mental state dimension. For example, in the three-dimensional social presence model proposed by Gunawardena^19^, two dimensions express psychological states, including affect or emotional expression and psychological belongingness, and in the three-dimensional social presence model proposed by Nowak^39^, one dimension expresses the psychological state, namely, a deep sense of psychological involvement. Biocca^27,28^ further considered emotion as one of the important components of social presence. The mental state dimension further includes perceived message comprehension, level of attention allocation, emotional comprehension, and degree of emotional dependence. Among them, perceived message comprehension refers to an individual’s ability to understand the message of the interacting object and the individual’s perception of his or her ability to understand the message^26^. The level of attention allocation is the amount of attention that the individual allocates to the interaction object and the perceived amount of attention that the other individual allocates to the individual itself. The salience of social presence can be demonstrated by the level of attention allocation^41^. Affective comprehension refers to the individual’s ability to understand the emotional and attitudinal states of the interaction object, as well as the perceived ability of the other individual to understand the individual’s own emotions, which also includes empathy^27,28^. The degree of affective dependence refers to the extent to which the individual’s mental and attitudinal states influence and are influenced by the mental and attitudinal states of the interaction partner^26^.

The third dimension is presence with behavioural involvement and interaction, which is the deeper perception brought about by the mutual influence of the behaviours of the interacting parties. Kuwabara^42^ argues that in addition to spatial copresence with others and judgements of others’ identities, intentions, and attention, social presence also includes deeper interactive perceptions, such as a sense of behavioural participation. Biocca and Harms^27,28^ also suggest that behavioural participation is one of the important components of social presence, including behavioural interaction, mutual aid, and interdependence. The two scholars later further defined this dimension as the behavioural interdependence ability, i.e. the degree to which the behaviour produced by an individual influences and is influenced by the behaviour of the interacting object^29^.

In addition to these three dimensions, some scholars have proposed that social presence also contains a conscious presence-perception dimension, i.e. the individual’s experience of perceiving and being perceived by others as felt through interaction^43^. Different from the physical presence dimension, the conscious presence-perception dimension emphasizes feeling, while the former focuses more on objective presence. Harms et al.^26^ argue that social presence should include symmetrical perceptions corresponding to the individual’s subjective perceptions, i.e. whether the individual perceives the presence of the interacting object at the same level of consciousness as the other individual and whether the other individual perceives the presence of the interacting object at the same level as the corresponding individual. Nario-Redmond et al.^44^ describe presence perception in terms of co-presence awareness and argue that it is an important constituent dimension of social presence. Co-presence awareness refers to the degree to which an individual believes that the interaction object is not isolated, the degree to which the individual is peripherally or focally aware of others, and the individual’s sense of the degree to which others are peripherally or focally aware of him or her. Table 2 below is summary of the social presence dimension in previous studies.

**Table 2:** Summary of the social presence dimension in previous studies (human–human interactions).

Model dimension	Dimensional explanation	References
Superficial physical presence	It refers to the individual’s discovery and awareness the copresence of another person’s body, which is a physical level, objective presence resulting from the meaning that will inevitably arise from human physical attributes	^40^
	The conditions for generation are the presence of common environmental factors (copresence with others in space) and mutual recognition between individuals	^27,28^
	Presence is revealed through interaction or open communication in the environment	^42^
Presence of mental states	It is the subjective perception of people, including the expression and understanding of emotions, changes in attention distribution, and changes in mental state	^44,42,19^
	Attentional allocation addresses the amount of attention the user allocates to and receives from an interactant Perceived message understanding is the ability of the user to understand the message being received from the interactant, as well as their perception of the interactant’s level of message understanding	^41,29^
	Perceived affective understanding is the user’s ability to understand an interactant’s emotional and attitudinal states, as well as their perception of the interactant’s ability to understand the user’s emotional and attitudinal states	^26^
	Perceived affective interdependence is the extent to which the user’s emotional and attitudinal state affects and is affected by the emotional and attitudinal states of the interactant	^27,28^
	Refers to the perception of mutual attention, empathy, and mutual understanding between interacting objects Including the judgement of the identity, intention and attention of others, emotional expression and a sense of psychological belonging	^33,39^
Presence with behavioural involvement and interaction	The degree to which the behaviour produced by an individual influences and is influenced by the behaviour of the interacting object	^27,28,44,42^
Conscious presence	It is a deep perception brought about by the mutual influence of the actions of both parties in the interaction	^29^
	Includes behavioural interaction, mutual aid and interdependence	^27,28^
	It refers to the experience of perceiving and being perceived by others that people feel through interaction	^43,44^
	Contains symmetric perception corresponding to the individual’s subjective perception, i.e. whether the individual has the same perception of the other individual with respect to the level of awareness of copresence with the other individual and the corresponding individual’s ability to perceive the presence of the interacting object	^26–28^
	The degree to which the observer believes he/she is not alone and secluded, their level of peripheral or focal awareness of the other, and their sense of the degree to which the other is peripherally or focally aware of them	^29^

### Research on social presence in the network environment

Social presence in interpersonal interaction arises from the direct interactions that individuals establish with others in the environment^45^ and the intimate connections that individuals make with others^26^, and robots can also generate social presence properties when artificial intelligence technologies help them exhibit social attributes and human-like social behaviours and establish social relationships with humans^46^. Social robots can exhibit social capabilities, such as sensing emotions, conversational communication, learning recognition, establishing or maintaining social relationships, motor behaviour, and personality expression^15^, and possess anthropomorphic behaviours, such as self-determination, care, companionship, communication, and entertainment, based on the environment and, thus, can be considered to have social attributes. Robots with anthropomorphic behaviours and social attributes can socially interact with and be perceived their social presence by human individuals. The ultimate goal of human–robot interaction is to provide a strong social interaction experience^14^, and the higher the level of social presence, the more likely the robot is to be perceived as a real social character, the richer the sense of the human interaction experience^15^, and the more positive the perception of robots. However, there is a lack of theoretical research about robots’ social presence. The present study has been conducted on the social presence theory of human interaction^16^ and measurement models (^29,44,42^, social robot behaviour and function^15^, human–robot interaction mechanism studies^14,15^), and other related studies to explore robots’ social presence from the perspective of social attributes.

## Research model

### Theoretical framework

**Figure 1:**
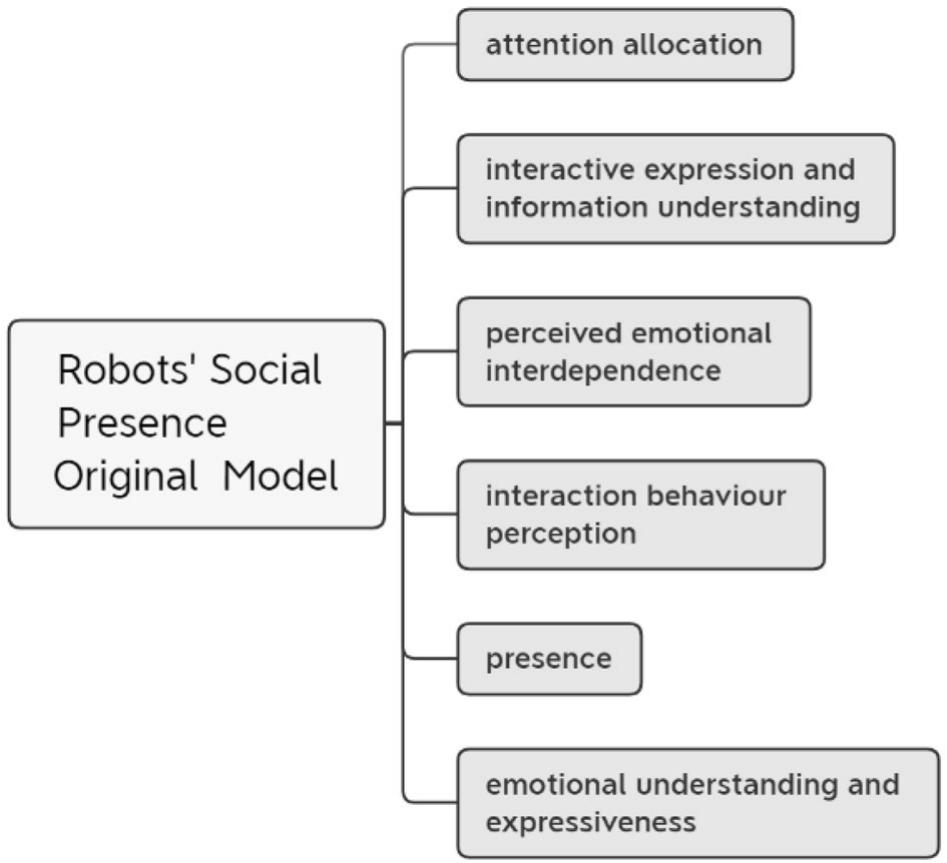
Original model of robots’ social presence.

This study proposes a definition of robots’ social presence, which means that in the case of direct interaction between a human individual and a social robot with artificial intelligence, if the individual can perceive the same or similar feelings as when interacting with a real human, then the latter is considered to have social presence. Based on the four dimensions of social presence in human interaction summarized above, this study proposes a six-dimensional model for robots’ social presence, including attention allocation, interactive expression and information understanding, emotional understanding and expressiveness, perceived emotional interdependence, perceived degree of interaction behaviour, and presence. The original model of the study is shown in Fig. 1.

Of these, the first four dimensions directly reference the secondary dimensions of the mental state social presence dimension in human–computer interactions. Because the key factors affecting the level of attention allocation, perceived message comprehension, emotional comprehension, and degree of emotional dependence in the psychological dimension of human–robot interaction differ, all four secondary dimensions are included as separate subdimensions of the robots’ social presence. Of the human–robot interaction dimensions, attention allocation^41^ refers to the amount of attention an individual can allocate to the robot and the amount of attention the individual perceives the other person is allocating to him or herself. Interactive expression and information understanding^47^ refers to the degree to which the individual perceives that both parties to the interaction understand the information in the process. Emotional understanding and expression^48^ refers to the ability of the individual in the interaction to understand the emotions and expressions of the robot and the ability of the individual to perceive the expression of the robot’s emotional and attitudinal states. Perceived emotional interdependence^49^ refers to the extent to which an individual’s emotional and attitudinal states influence and are influenced by the robot’s emotional and attitudinal states. Furthermore, social presence in interpersonal interaction includes two main dimensions, i.e. physiological presence and copresence perception^26^. In the context of human–robot interactions, the former refers to the individual’s ability to perceive the copresence of the robot in a space after interacting with it through vision, hearing, etc. while the latter refers to the significant presence of the robot as perceived by the individual subjectively and consciously through the interaction. Of these, the latter is the basis for the generation of the former, and it is difficult to distinguish between the two, so this study proposes the presence dimension, which measures both the individual’s perception of the presence and copresence of the physical dimension of the social robot in the interaction.

Finally, this study directly references the presence dimension of behavioural engagement and interaction in interpersonal interactions and proposes the perceived degree of interaction behaviour dimension of robots’ social presence^50^, which refers to the degree to which individuals perceive behavioural influence on and are influenced by the robot’s behaviour.

## Research methodology

### Study 1 design the scale of robots’ social presence

Using social presence as the key word, we retrieved 611,000 related papers in Google Academic database and screened out the scales related to social presence, such as Network psychometric scale^26^, interpersonal social existence theory^51^, and structural dimensions of existence and reality judgment in virtual environments^52^. We combined items with similar meanings and 105 questions were obtained. After evaluation by three experts, we selected 24 questions related to human–robot interaction from the scale of human interaction and virtual interaction. Finally, according to the advice of experts, the 24 questions were divided into 6 dimensions to form the initial scale shown in Table 3. All items in the scale are administered in English. They were translated into Chinese using the translate-retranslate method. The actual survey was conducted in Chinese.

**Table 3:** The original scale of robots’ social presence.

Dimension	Number	Item	References
Perceived presence	1	During the interaction with the social robot, I noticed it	^29,52^
	2	The social presence of robots is obvious to me	
	3	I think social robots receive my attention in interactions	
	4	I think that when interacting with robots, we co-exist in the space in the present moment	^53^
Interaction behaviour perception	5	It helped me in the process of interacting with the social robots	^27^
	6	I was able to work with social robots to complete a job	
	7	The behaviour of a social robot is based on my behaviour	^29,33^
	8	The behaviour of a social robot during an interaction is closely related to my behaviour	
Interactive expression and information understanding	9	I do not think the social robot understands my expressions when I interact with it	^29,33^
	10	I think I am able to clearly express my thoughts to a social robot*	
	11	I think social robots are able understand my thoughts correctly	
	12	I think I am able to communicate with social robots through language	
Perceived emotional interdependence	13	I think the social robots in our interactions affected how I felt	^26,30^
	14	The mood of the robot affects the mood of our interaction*	
	15	The attitude of the robot during the interaction influenced my feelings	
	16	I felt the atmosphere between us during the interaction	
Attention allocation	17	It is easy for me to become distracted from interacting with the robot when other things are going on	^27,29^;
	18	Social robots are easily distracted when interacting	
	19	When interacting with a social robot I think it is watching my actions too*	
	20	I keep an eye on the social robot as I interact with it	
Emotional understanding and expressiveness	21	I am well aware of the emotions of social robots*	^27,29^;
	22	I think social robots can sense my emotions*	
	23	I think social robots show emotions when they interact with me*	
	24	We can correctly interpret each other’s emotions*	^19^

The whole scale is the initial scale developed according to the initial model.

*indicates that the questions deleted after three rounds of survey.

Non *questions are the items left after deletion.

**Figure 2:**
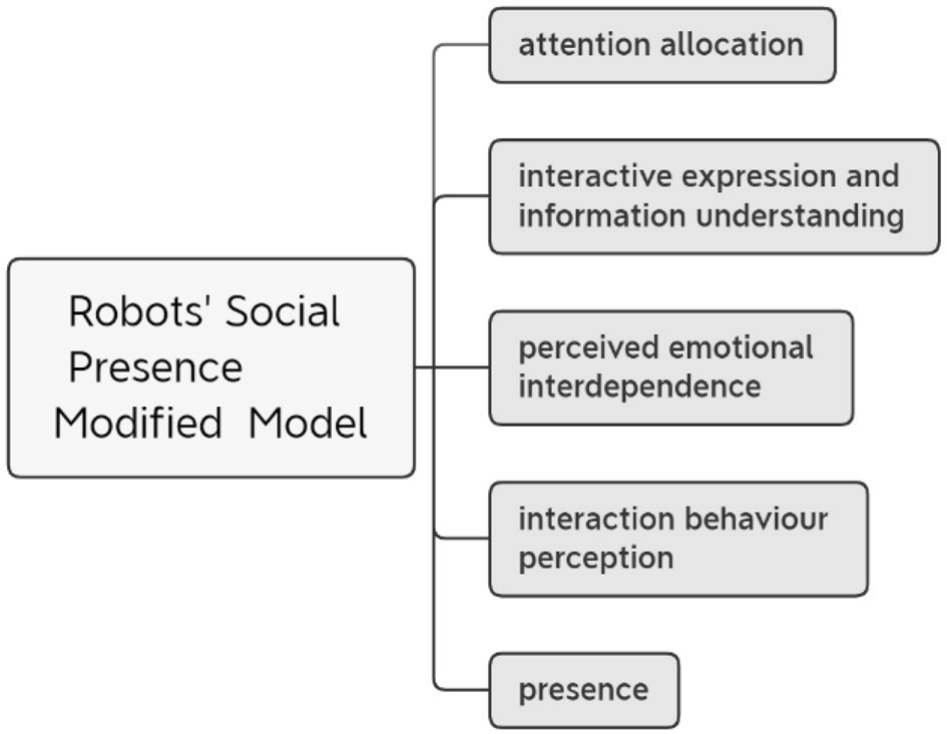
Modified model.

Based on the theoretical framework of robots’ social presence, this study will design and develop the corresponding scales. The scale development construction consists of three studies. The first study used the expert evaluation method and user interviews to evaluate and revise the original model and the measurement scale to form the final model (Fig. 2) and the revised scale (Table 4). The second study distributed the modified scale and collected 93 valid questionnaires. The data analysis results showed that the model had high reliability and some basic structural validity, the model fit was further improved after the revision of the question items, and the final model was determined. In the third study, the final model questionnaire was distributed and 494 valid questionnaires were collected, and the analysis results proved that the scale had good reliability and a high validity of fit. The 5-dimensional robot social presence model was finalized, and a 17-question questionnaire scale was developed.

**Table 4:** Basic information for the respondents of the pilot questionnaire survey.

Measurements	Alternatives	Frequencies	Percentage
Gender	Male	19	20.43%
	Female	74	79.57%
Age	< 18	1	1.08%
	18–25	76	81.72%
	26–30	13	13.98%
	31–40	2	2.15%
	41–50	1	1.08%
	> 51	0	0.00%

### Study 2 expert evaluation and user interviews

In this study, first, three experts were invited to evaluate the original model and scale validity of robots’ social presence. They were from the School of Economics and Management and the School of Information Science and Technology of Beijing University of Chemical Technology, including one expert in the field of artificial intelligence, one expert in the field of psychology, and one expert in the field of sociology. Each expert was required to evaluate the definition and model of robots’ social presence proposed in this study, the expert tested the face validity of the scale to measure the relationship between items and Robot’s Social Presence, and to review the content validity and discriminant validity of each dimension of the scale individually.

The three experts believe that the definition proposed in this study provides a complete overview and representation of robot social presence in human–robot interaction from the following three perspectives: interaction object, interaction mode, and interaction outcome. However, currently humans understand the emotional expression of social robots mainly through recognizing facial expressions^54^, according to the uncanny valley theory^55^, humans do not like robots with overly anthropomorphism in appearance, so common social robots usually have a low degree of facial anthropomorphism and a single expression design, which leads to the human recognition of robot emotions being generally low, and the emotional expression ability of social robots cannot be effectively measured by questionnaires. At the same time, the interpretation of emotional expressions is not the main factor affecting the social presence of robots under current technological conditions and human acceptance levels. Therefore, the discriminant validity of the dimension of emotional understanding and expression ability and the two dimensions of perceived emotional interdependence in the original model is low. Respondents have difficulty distinguishing between the meanings of the questions in the two dimensions due to the limitations of the technical conditions and personal experience, and a dimensional merger is recommended.

After that, this study invited five respondents with experience in using social robots to conduct structured interviews, they were asked to fill out the original questionnaire based on their own experience and to assess whether the language of the questions was clear and precise enough, whether there was repetition or conflict in the understanding of the question items, and whether the meaning was concise and easy to understand. The questions on the comprehension and expressiveness dimensions were questioned. They indicated that the social robots they could come across in their daily lives generally had fixed facial expressions, showed a single type of emotion, and fluctuated to a small degree, and the anthropomorphic emotional expression felt during interaction was not significant, so they rarely paid attention to the robot’s emotional expression, and it had a very limited impact on the interaction effect. On the other hand, two respondents believed that their needs regarding the hardware functions of social robots were greater than their needs for emotional communication, and they could not imagine a scenario of interacting with a social robot with anthropomorphic emotions and language expressions, so they could not make accurate judgements, and this dimension in the questionnaire was not an important dimension for assessing the social presence of robots. The results of the expert evaluation and user interviews were combined, related questionnaire items on the emotional understanding and expression ability dimension was removed from this study, and a 5-dimensional modified model of robots’ social presence and the corresponding measurement questionnaire were proposed, as shown in Fig. 2.

### Study 3 pilot questionnaire survey

Based on the modified model and the corresponding scale-modified questionnaire, this study conducted a questionnaire survey. The questionnaire contained two parts. The first part was demographic questions about the respondent’s gender, age, and whether they had experience with social robots, and the second part focused on the modified scale of robots’ social presence with 19 questions, measured using a 5-point Likert scale, where 1 = strongly disagree and 5 = strongly agree. The questionnaire took approximately 2 to 5 min to complete.

#### Responders

In this study, 200 questionnaires were distributed, and 172 questionnaires were returned. Of these, 93 valid samples (i.e. responses with relevant experience) were obtained for this study based on whether or not they had experience with social robots, including 19 male individuals (20.43%) and 74 female individuals (79.57%), one of them (1.08%) under the age of 18, 76 individuals (81.72%) between the ages of 18 and 25, 13 individuals (13.98%) between the ages of 26 and 30, 2 individuals (2.15%) between 31 and 40 years old, and 1 individual (1.08%) over 40 years old. The specific basic information of the respondents is shown in Table 4.

#### Ethical approval

All methods were performed in accordance with relevant guidelines and regulations. All study protocols were approved by the institutional review board of the Ethics Committee of Beijing University of Chemical Technology. Informed consent was obtained from all the participants.

#### Data analysis

Exploratory factor analysis (EFA) was first performed to confirm the discriminant validity of the model dimensions. The results of the consistency analysis indicated good reliability of the questionnaire (Cronbach’s alpha = 0.807 > 0.800). The results of Bartlett’s sphericity test indicated the presence of a common factor (χ^2^ = 879.382, d***f*** = 171, ***p*** < 0.01). The results of the Kaiser–Meyer–Olkin index analysis indicated a high correlation between the factors (KMO = 0.807 > 0.800), which indicates that the questionnaire data are suitable for exploratory factor analysis^56^. The exploratory factor analysis results showed that the five dimensions of presence, attention allocation, interactive expression and information understanding, degree of emotional-attitudinal interaction, and perceived dimension of interaction behaviour in the modified model explained 66% of the variance, indicating the strong explanatory validity of the model^57^. The specific analysis results are shown in Table 6.

Using 0.5 as the criterion for factor loading extraction, the item “I think the social robot is also paying attention to me when interacting with it” had an action factor loading of less than 0.5 and was not classified in any of the dimensions, indicating that the question item was of low quality, so it was not considered in the next analysis. Exploratory factor analysis was conducted again to gain construct validity, and the question items in the principal component matrix were reasonably distributed over 5 factors, which was consistent with the designed 5-dimensional model. The specific analysis results are shown in Table 5.

**Table 5:** Construction validity analysis of the pilot questionnaire survey.

Dimension	Number	Items	Factor loading									
			1	1^#^	2	2^#^	3	3^#^	4	4^#^	5	5^#^
Perceived Presence	3	I think social robots receive my attention in interactions	0.787	0.787								
	5	I think that when interacting with robots, we co-exist in the space of the present moment	0.771	0.782								
	2	The social presence of robots is obvious to me	0.739	0.746								
	20	I keep an eye on the social robot as I interact with it	0.707	0.727								
	1	During the interaction with the social robot, I notice it	0.574	0.585								
	6	I was able to work with social robots to complete a job			0.825	0.808						
Interaction behaviour perception	7	The behaviour of a social robot is based on my behaviour			0.759	0.790						
	5	It helped me in the process of interacting with the social robots			0.714	0.744						
	8	The behaviour of a social robot during an interaction is closely related to my behaviour			0.703	0.670						
	11	I think social robots are able to understand my thoughts correctly					0.825	0.828				
Interactive expression and information understanding	10	I think I am able to clearly express my thoughts to a social robot					0.774	0.807				
	12	I think I am able to communicate with social robots through language					0.595	0.599				
	13	In our interactions, I think the social robots affected how I felt							0.898	0.893		
Perceived emotional interdependence	15	The attitude of the robot during the interaction influenced my feelings							0.816	0.818		
	16	I felt the atmosphere between us during the interaction							0.660	0.667		
	18	Social robots are easily distracted when interacting									0.793	0.791
Attention allocation	17	It is easy for me to become distracted from interacting with the robot when other things are going on									0.761	0.774
	9	I do not think the social robot understands my expressions when I interact with it									0.714	0.712
	19	When interacting with a social robot, I think it is watching my actions too*										—

*indicates the item that was deleted due to action factor loading less than 0.5 in the first exploratory factor analysis.

^#^indicates the results of the exploratory factor analysis after removing the item*.

In the rotated component matrix, the item “I pay attention to the social robot when interacting with it” has a factor loading less than 0.5 in the dimension of attention allocation dimension and a factor loading greater than 0.5 in the dimension of perceived presence, indicating that the item better explains the presence dimension and can be classified as a presence dimension^58^. In addition, five respondents were randomly contacted in this study, and they gave feedback that when interacting with a social robot, they only paid attention to the robot when the robot displayed social behaviours that caught their attention and made them feel like they were being with the robot. Moreover, when the researchers explained the concepts of presence and attention allocation dimensions, they all agreed that the question item better explained the perceived presence of the social robot. Therefore, in the next analysis this question item was listed as the presence perception dimension.

In addition, the item “I think it is difficult for the social robot to understand me” has a factor loading of less than 0.5 in the interaction expression and information comprehension dimensions and a factor loading of more than 0.5 in the attention allocation dimension, indicating that this item is applicable for explaining the attention allocation dimension. According to the results of the postquestionnaire interviews, the respondents’ have different understandings between the describe for “not understanding” in the item and the reason for social robots giving false feedback during the interaction. According to the experiences of all five respondents, social robots are prone to slow recognition, slow feedback, or even mishearing the user’s task and giving completely wrong feedback when faced with continuous voice commands, which they attribute to that social robots do not understand commands. In fact, the reason for this phenomenon is that social robots receive information too often, making the reflection slow or missing. The problem belongs to the level of robots’ intelligence. Respondents agreed that the word “unintelligible” in the question was too vague and could lead to ambiguity and different result tendencies and suggested deleting it. However, in the exploratory factor analysis, the question item had a clear division of factor components, and the factor loadings were 0.712 and greater than 0.5. Therefore, the question item was temporarily retained in the next analysis, and the results of the validation factor analysis were used to determine whether to delete the question item.

Based on the results of the validation factor analysis, the results of each model fit metric had acceptable fit, including chi-square/degree of freedom (χ^2^/d***f***) = 1.333, root mean square error of approximation (RMSEA = 0.061), Tucker-Lewis index (TLI = 0.928), comparative fit index (CFI = 0.943), fitness test (GFI = 0.845), root mean squared of asymptotic residuals (RMSEA = 0.061), and standardized residuals mean squared (SRMR = 0.087). Moreover, the canonical fit index (NFI = 0.812) and the adjusted goodness-of-fit index (AGFI = 0.781) were close to the acceptable range. These results indicate that the model fit is good^59^, but further optimization is needed. The results of the specific analysis are presented in Table 6.

**Table 6:** Modified model fit of both the pilot and final questionnaire survey.

Standards	Pilot survey*	Pilot survey**	Final model	Acceptable	Excellent
Minimum fit function chi-square (χ^2^)	161.241 (p < 0.001)	131.294 (p < 0.001)	222.432 (p < 0.001)		
Degrees of freedom (d***f***)	121	103	103		
χ^2^/d***f***	1.333	1.275	2.160	< 5.00	< 3.00
GFI	0.845	0.875	0.950	> 0.80	> 0.90
RMSEA	0.061	0.055	0.048	< 0.08	< 0.05
AGFI	0.781	0.787	0.926	> 080	> 0.90
NFI	0.812	0.841	0.939	> 0.90	> 0.95
CFI	0.943	0.959	0.966	> 0.90	> 0.95
TLI	0.928	0.946	0.955	> 0.90	> 0.95
IFI	0.946	0.961	0.966	> 0.90	> 0.95
SRMR	0.087	0.066	0.052	< 0.09	< 0.08

*indicates the results of the factor analysis after removing the question “I think the social robot is also paying attention to my actions when I interact with it”.

**indicates the results of the factor analysis after deleting the question “I think social robots have a hard time understanding my words”.

According to the results of the consistency analysis, the Cronbach’s alpha values of all dimensions of the model were greater than 0.6, indicating that the questionnaire had high reliability. According to the results of the convergent validity analysis, the factor convergent validity of the attention allocation dimension was low, the average variance extracted (AVE) = 0.431 < 0.5, and the factor loading of the item “I think it is difficult for social robots to understand me” was 0.499, which was lower than 0.5. Combined with the results of the structured interview in Study 1, it was concluded in this study that this question item differed significantly from the other questions in the dimension, so it was removed from the scale and not explored in future data analyses.

The construct reliability (CR) values for the other four dimensions of the model were greater than 0.8, indicating that the questions in the dimensions explained the variable in a largely consistent manner. The average variance extracted (AVE) values were all greater than 0.5, indicating that the model had good convergent validity across the dimensions.

Adjusting for the two items resulted in the final robots’ social presence scale for this study, which contained 17 items. The results of the validation factor analysis indicated that the model had a high fit and outperformed the modified model, including χ^2^/d***f*** = 1.275, RMSEA = 0.055, TLI = 0.946, NFI = 0.841, and CFI = 0.959, as shown in Table 6. In the validation factor analysis, the factor loadings of each item were greater than 0.5, the CR of each dimension was greater than 0.6, and the AVE was greater than 0.5, which met the acceptable criteria and indicated that the final scale convergent validity was met. The specific results of the analysis are shown in Table 7.

**Table 7:** Study 2: Scale validity analysis of the pilot questionnaire survey.

Constructs	number	Items	Internal reliability		Convergent validity		
			Item	Cronbach’s alpha	Factor load	CR	AVE
Perceived presence	1	During interaction with the social robot, I noticed it	5	0.851	0.652	0.850	0.535
	2	The presence of social robots is obvious to me			0.790		
	3	I think social robots receive my attention in interactions			0.825		
	4	I think that when interacting with robots, we co-exist in the space of the present moment			0.676		
	20	I keep an eye on the social robots as I interact with them			0.699		
Interaction behaviour perception	5	It helped me in the process of interacting with the social robots	4	0.850	0.684	0.839	0.569
	6	I was able to work with social robots to complete a job			0.683		
	7	The behaviour of a social robot is based on my behaviour			0.788		
	8	The behaviour of a social robot during an interaction is closely related to my behaviour			0.849		
Interactive expression and information understanding	10	I think I am able to clearly express my thoughts to a social robot	3	0.803	0.822	0.823	0.614
	11	I think social robots are able to understand my thoughts correctly			0.906		
	12	I think I am able to communicate with social robots through language			0.462		
Perceived emotional interdependence	15	The attitude of the robot during the interaction influenced my feelings	3	0.749	0.601	0.821	0.610
	13	I felt the atmosphere between us during the interaction. I think the social robots in our interactions affected how I felt			0.856		
	15	The attitude of the robot during the interaction influenced my feelings			0.859		
Attention allocation	17	It is easy for me to become distracted from interacting with the robot when other things are going on	2	0.703	0.844	0.685	0.529
	16	Social robots are easily distracted when interacting			0.589		

### Study 3 final questionnaire design

To verify the stability of the final robots’ social presence scale across samples, the study was conducted again with 719 questionnaires. Again, 494 valid questionnaires were obtained based on whether they had experience with social robots as a screening criterion. These included 174 male respondents (35.22%) and 320 female respondents (64.78%). Seven respondents (1.42%) were under 18 years old, 323 respondents (65.38%) were between 18 and 25 years old, 46 respondents (9.31%) were between 26 and 30 years old, 42 respondents (8.50%) were between 31 and 40 years old, 38 respondents (7.69%) were between 41 and 50 years old, and 38 respondents (7.69%) were between 51 and 60 years old (7.69%).

The results of the consistency analysis indicated the good reliability of the questionnaire (Cronbach’s alpha = 0.900 > 0.800). The results of Bartlett’s sphericity test indicated the presence of common factors (χ^2^ = 3576.290, d***f*** = 136, ***p*** < 0.01). The results of the Kaiser–Meyer–Olkin index analysis indicated a high correlation between the factors (KMO = 0.883 > 0.800). These results indicate that the questionnaire data are suitable for exploratory factor analysis.

The results of the exploratory factor analysis indicated that the model extracted five factors, and each factor contained question items consistent with the final robots’ social presence scale. The specific results of the analysis are shown in Table 8.

**Table 8:** Exploratory factor analysis of the final questionnaire survey.

Number	Item	Dimension					Reliability		Convergent validity		
		1	2	3	4	5	Item	Cronbach’s alpha	Factor load	CR	AVE
3	I think social robots receive my attention in interactions	0.740					5	0.843	0.668	0.845	0.522
20	I keep an eye on the social robot as I interact with it	0.725							0.703		
4	I think that when interacting with robots, we co-exist in the space of the present moment	0.723							0.787		
1	During interaction with the social robot, I notice it	0.720							0.727		
2	The presence of social robots is obvious to me	0.684							0.721		
5	It helped me in the process of interacting with the social robots		0.742				4	0.815	0.691	0.809	0.516
7	The behaviour of a social robot is based on my behaviour		0.741						0.661		
8	The behaviour of a social robot during an interaction is closely related to my behaviour		0.696						0.755		
6	I was able to work with social robots to complete a job		0.655						0.761		
13	I think the social robots in our interactions affected how I felt			0.811			3	0.814	0.769	0.814	0.594
15	The attitude of the robot during the interaction influenced my feelings			0.789					0.803		
16	I felt the atmosphere between us during the interaction			0.661					0.739		
11	I think social robots are able to understand my thoughts correctly				0.810		3	0.789	0.642	0.798	0.571
10	I think I am able to clearly express my thoughts to a social robot				0.788				0.817		
12	I think I am able to communicate with social robots through language				0.698				0.796		
18	Social robots are easily distracted when interacting					0.831	2	0.703	0.794	0.705	0.546
17	It is easy for me to become distracted from interacting with the robot when other things are going on					0.830			0.679		

The results of the validation factor analysis showed that the model had a high fit, including χ^2^/d***f*** = 2.160, RMSEA = 0.048, TLI = 0.928, NFI = 0.939, AGFI = 0.926, SRMR = 0.052, CFI = 0.966, GFI = 0.950. The results of the specific analyses are shown in Table 6. In the validation factor analysis, the Cronbach’s alpha of each dimension was greater than 0.7, indicating that the dimensions have high internal consistency and that the scale has high reliability. The factor loadings of each item were greater than 0.6, and the CR of each dimension construct reliability was greater than 0.7, indicating that the questions consistently explained the dimension to which they belonged. The average variance extracted AVE of each dimension was greater than 0.5, indicating that the dimensions all had high convergent validity. The factor convergent validity of each dimension of the scale was verified. The specific analysis results are shown in Table 8.

### Informed consent

Informed consent was obtained from all the participants and/or their legal guardians.

## Discussion

In this study, the properties and social presence of robots from the perspective of social interactions are explored, the definition of robots’ social presence is given, and model construction is conducted. The previous studies on social robots have focused on the intelligence level and functional properties of robots, and few social properties of social robots have been explored from a social perspective. In the social presence-related studies, researchers have mainly explored social presence in interpersonal interactions, and few studies have focused on the social presence properties of robots with artificial intelligence. This study synthesizes previous research theories on the concept, dimensions, and scales of interpersonal social presence, as well as social presence in online social networking-related studies, to explore the social presence of robots.

In their definition, Heeter and his colleagues^25^ consider social presence a feeling of being with others. This study argues that in human–robot interaction, this feeling is a human mental state and subjective perception that is generated by humans through direct interaction with a robot with human-like behaviour. In interpersonal social presence, previous researchers have argued that social presence contains the following four dimensions: superficial physical presence, the presence of mental states, presence with behavioural involvement and interaction, and conscious perception of presence. Considering the differences between human–robot interactions and interpersonal interactions in terms of the interaction object, interaction mode, and interaction outcome, this study proposes a model of robot social presence, i.e. the level of social presence of a robot can be judged by the perception of the significant of presence, the level of attention allocation, the perception of interactive expression and information understanding, the perception of emotional interdependence, and the perception of interaction behavioural interactions.

In the model construction, this study merges two dimensions of interpersonal social presence, i.e. the surface physiological presence and the conscious perception of presence, and proposes the presence dimension of robots’ social presence. According to the previous research, the social presence of humans is an inevitable attribute that is inherent in life, while robots’ social presence is created through interactions with humans after the development of technology to achieve deeper anthropomorphism and a higher level of intelligence. It is only when humans realize that the object of the interaction is a human-like social character with social capabilities that social robots are considered to have a social presence on this basis. It is difficult to make a clear delineation between the two dimensions in human–robot interaction, so they are merged into the presence dimension. In addition, three of the dimensions of the final model of robots’ social presence directly refer to the three secondary dimensions of the attention allocation, the perceived message understanding, and the degree of emotional dependence of the mental state social presence dimension in human interaction, i.e. attention allocation, interactive expression and information understanding, and perceived emotional interdependence.

Presence is generated by connections, and the presence of an interacting object generates behavioural enhancement support, increased attention, etc., which establish the first connections for interaction in the human brain^60^. Attention-related changes have been found to be closely related to the neural effects of social presence in studies measuring changes in social task-related responses. In human–computer interactions, the degree of human attentional change due to the interaction can reflect the significant degree of the human’s perception of robot presence, and changes in attentional allocation are an important dimension reflecting the perception of social presence. Short et al.^45^ argued that social presence describes the degree of communication and information exchange through which interactants can convey information, express emotions, and establish and develop relationships.

In human–robot interactions, the ability of both interacting objects to transmit information in real time and the mutual perception of the accessibility of the transmission can measure the quality of the interaction. The speed with which the robot receives information and the quality of the feedback affects the human’s judgements about the degree of robot humanity and the effectiveness of the interaction, which in turn affects the human’s judgements about the salience of social presence. Interactors can also perceive each other’s emotional responses through interaction. Emotional displays and interactive responses are key to prompting human–robot interaction, and as human interest in human–robot interaction increases, there is growing interest in the ways in which robot intelligence and emotion can be implemented, with the expectation of enhancing emotional communication and maximizing the social response of the interacting object^61^. Interactors feel higher levels of mutual knowledge, understanding, and greater empathy, which in turn leads to strong psychological dependence, so that emotional interaction deepens the level of social presence felt by the person, and the degree of emotional interdependence is one of the key factors affecting the level of robot presence.

In this study, an original model of six-dimensional robots’ social presence was proposed, the measurement dimensions were revised based on expert evaluation and user interviews on the characteristics of social robots, and the emotional understanding and expression dimensions were removed^49^ to propose a final model with five dimensions. In the interpersonal social presence measurement model of Nario-Redmond et al.^44^ and the network social presence model of Biocca^27,28^, the emotional expression and understanding is one of the important manifestations of the presence of mental states in the interactor’s level of presence, and the level of interaction deepens from surface physical presence to the presence of mental states through emotional expression and empathic perception. Because the inherent physiological attributes of human interactors in human interactions allow them to achieve physiological emotional expression and relatively accurate psychological emotional understanding, in the human–computer interaction environment, robots mainly express emotions through information such as facial expressions, behavioural gestures, and voice intonation^62^, with facial expressions being the most direct and effective way to express emotional information^63^. The degree of anthropomorphism and the level of intelligence of a social robot determine the accuracy of the interactor in recognizing and understanding the robot’s emotions.

Experts believe that the technical level of social robots cannot achieve enough anthropomorphic facial expressions to serve as the most important way for human users to identify emotional expressions, and that the development and design of social robots is limited by the level of human acceptance of humanoid robots, which weakens the processing of facial expressions and other emotional expressions, and that human judgement of robot emotions is vague and the recognition accuracy is low; thus, it cannot accurately reflect the robot’s level of social presence. The results of user interviews show that robots are generally unable to display complex facial expressions, have a single voice tone, lack human-like emotional ups and downs, and display fewer types of emotions in daily interactions. Meanwhile interactors tend to ignore the robot’s emotions in interactions or directly assume that the robot does not have emotions, but the lack of emotions in the design does not affect the effectiveness of the human–robot interactions, suggesting that robot’s emotional expression is not a key factor affecting the robots’ social presence.

The scale in this study measured only two basic questions after censoring the measurement questions in the interaction expression and information comprehension dimension, which can be supplemented with refined questions in the subsequent targeted discussion of the perceived degree of comprehension of social robot-subject interactions to facilitate further, targeted measurement of robots’ social presences. At last, in further study, in view of the differences between the way Asian countries treat technology and western cultures, which will lead to the differences in the understanding of the scale by western cultures, the scale will also be tested across cultures.

## Conclusions

Based on theories and concepts related to human interaction and social presence in a networked environment, this study aims to propose a definition of social robot social presence, construct a model, and propose a corresponding measurement scale. This study defines robots' social presence, i.e. in the case of human individuals interact directly with a social robot with artificial intelligence, if the individual can perceive the same or similar feelings as when interacting with a real human, then the latter is considered to have social presence. In this study, a robot social presence model containing the following five dimensions was constructed: presence, attention allocation, interactive expression and information understanding, perceived emotional interdependence, interaction behaviour perception. The corresponding 17-question measurement questionnaire was obtained, and the scale was validated.

In the expert evaluation and user research, the six dimensions of the original model were revised, and the emotional understanding and expression dimensions were deleted based on the current situation analysis of experts regarding the emotional expression of social robots and the human emotion judgement ability and the investigation of the individual’s acceptance of the robot’s emotional expression and functional requirements during the interaction. The revised model dimensions were verified in the following data analysis. The results of the two rounds of questionnaire research showed good reliability of the measurement questionnaire, high inter-factor correlation, high model fit, high internal consistency of the dimensions, high reliability of the scale, and high convergent validity of all dimensions.

This study provides a conceptual specification and measurement tool of the social attributes of robots for researchers related to social robots and provides a theoretical basis and measurement scale for further explore the improvement of the quality of human life and the effectiveness of human–robot interactions with social robots. In future research, this study will be further refined and the limitations of the scale will be explained. First, we need a larger range of people of different age levels and education levels to participate in the study. Second, how to measure the effect of emotional dependence on the level of robots’ social presence in human–computer interaction under the existing conditions may require specific experimental sessions for obtaining the measurement. At last, in further research, we will expand the size and audience of the study and expect further extensions to the existing measurement tools.

## Data Availability

The datasets generated during and/or analysed during the current study are available from the corresponding author on reasonable request.
